# Supplementary material for: The diagnostic yield of exome sequencing in liver diseases from a curated gene panel
Source: Sci Rep. 2023 Dec 6;13:21540. doi: 10.1038/s41598-023-42202-1 (PMC10700603; doi:10.1038/s41598-023-42202-1)
Supplement: Supplementary file 4 — Supplementary Information 4. [file 41598_2023_42202_MOESM4_ESM.docx]

**Supplementary material:**

**Supplementary figures:**

Supplementary Figure 1: The search and establishment of a list of liver genes

**Supplementary tables:**

Supplementary Table 1: The original research of the samples used in this study.

Supplementary Table 2: The inheritance mode and biological annotations of 502 genes related to Mendelian disorders and liver phenotypes

Supplementary Table 3: Clinical characteristics and genetic testing information for two cohorts.

Supplementary Table 4: Stepwise filtering to identify individuals with pathogenic/likely pathogenic variants in liver genes.

Supplementary Table 5: Effects of minor allele frequency filtering on the burden of candidate pathogenic variants in genes associated with chronic liver diseases

Supplementary Figure 1: The search and establishment of a Mendelian liver disease gene list

HPO

n=954

OMIM

n=609

Orphanet

n=1192

Literature review

n=24

liver gene search

Remove duplicates

Gene list for curation

n=959

Exclusion

n=457

liver gene list

n=502

Keywords or phrase used to search for liver gene list: "liver", "biliary", "hepa", "cirrhosis", "Reye syndrome", "transaminases", "gallbladder", "cholestasis", "jaundice", "bile", "cholecystitis", "cholelithiasis", "portal", "steatosis", "dyslipidemia", "hyperlipidemia", "hypercholesterolemia", "hypertriglyceridemia", "insulin resistance", "hepatic fibrosis", "hypobetalipoproteinemia", "abetalipoproteinemia", "glycogen storage diseases", "lysosomal storage disorders", "lysosomal acid lipase deficiency", "fatty acid oxidation", "Gaucher’s", "lipodystrophies", "polycystic ovarian syndrome", "hemochromatosis",

Supplementary Table 1: The original research of the samples used in this study

| Original research | Current study population | n=10, 801 | |
| --- | --- | --- | --- |
| WHICAP | Population-based study of Medicare recipients aged ≥ 65 years in northern Manhattan | 2983 | 27.6% |
| Genetics of neurological disorders, OCD, ALS, Epilepsy, memory, schizophrenia | Self-reported healthy family members of patients with epilepsy, ataxia, neuromuscular disorders, brain malformations, neurodevelopmental disorders, OCD, schizophrenia, ALS, early-onset Parkinson disease, and other neurological disorder | 2893 | 26.8% |
| Diagnostic sequencing study of undiagnosed genetic disorders | Self-reported healthy family members of patients with undiagnosed disorders from general genetic clinic | 1564 | 14.5% |
| 27 additional studies | Self-reported healthy family members or control participants to a variety of genetic studies | 325 | 3.0% |
| **Genetic study of chronic liver diseases** | Self-reported healthy family members to chronic liver disease genetic study | 94 | 0.9% |
| Genetics study of kidney and Genitourinary disorders | Patients with chronic kidney diseases or genitourinary disorders | 2187 | 20.2% |
| **Genetic study of chronic liver diseases** | Patients with chronic liver diseases | 758 | 7% |

WHICAP: Washington Heights-Inwood Columbia Aging project; ALS: amyotrophic lateral sclerosis; OCD: obsessive compulsive disorder.

Supplementary Table 2: The inheritance mode and biological annotations of 502 genes related to Mendelian disorders and liver phenotypes

| 502 liver genes | Hepatocyte  Intrinsic | Biliary | Metabolism | Immune | Development |
| --- | --- | --- | --- | --- | --- |
| AD (n=61) | 26 | 9 | 12 | 9 | 31 |
| AD& AR (n=62) | 26 | 12 | 26 | 16 | 33 |
| AR (n=363) | 161 | 53 | 180 | 60 | 224 |
| XLD (n=1) | 0 | 0 | 1 | 0 | 0 |
| XLR(n=15) | 6 | 2 | 3 | 5 | 10 |

AD: Autosomal dominant, AR: Autosomal recessive, XLD: X-linked dominant, XLR: X-linked recessive.

Supplementary Table 3: Clinical characteristics and genetic testing information for three cohorts

| Characteristics | Healthy control cohort (n=7856) | Proportion in healthy control cohort | Chronic kidney disease cohort  (n=2187) | Proportion in CKD cohort | Chronic liver disease cohort  (n=758) | Proportion in CLD cohort |
| --- | --- | --- | --- | --- | --- | --- |
| Gender |  |  |  |  |  |  |
| Male | 3176 | 40.4% | 945 | 43.2% | 351 | 46.3% |
| Female | 4680 | 59.5% | 1242 | 56.8% | 407 | 53.7% |
|  |  |  |  |  |  |  |
| Age at time of study entry |  |  |  |  |  |  |
| 0-21 yr | 240 | 3.1% | 278 | 12.7% | 255 | 33.6% |
| 22-44 yr | 1416 | 18.0% | 713 | 32.6% | 150 | 19.8% |
| 45-64 yr | 597 | 7.6% | 800 | 36.6% | 226 | 29.8% |
| ≥ 65 yr | 103 | 1.3% | 396 | 18.1% | 127 | 16.8% |
| Unspecified | 5500 | 70.0% |  |  |  |  |
|  |  |  |  |  |  |  |
| Self-declared race/ethnicity |  |  |  |  |  |  |
| White | 3427 | 43.6% | 1113 | 50.9% | 367 | 48.4% |
| Hispanic | 2303 | 29.3% | 435 | 19.9% | 136 | 17.9% |
| Black | 1070 | 13.6% | 330 | 15.1% | 95 | 12.5% |
| Asia | 420 | 5.3% | 224 | 10.2% | 61 | 8% |
| Other or unspecified | 636 | 8.1% | 85 | 3.9% | 99 | 13.1% |
|  |  |  |  |  |  |  |
| Sequencing Modality |  |  |  |  |  |  |
| Whole Exome Sequencing | 7735 | 98.4% | 2187 | 100% | 758 | 100% |
| Whole Genome Sequencing | 121 | 1.6% |  |  |  |  |
|  |  |  |  |  |  |  |
| Exome Capture Kit |  |  |  |  |  |  |
| Roche | 5954 | 76.7% | 1495 | 68.4% | 221 | 29.2% |
| IDTERPv1 | 1147 | 14.8% | 692 | 31.5% | 537 | 70.8% |
| Other | 664 | 8.6% |  |  |  |  |

Supplementary Table 4: Stepwise filtering to identify individuals with likely pathogenic variants in liver genes

| **Total WES**  **(n=10,804)** | **Healthy control cohort**  **(n=7856)** | **Kidney disease cohort**  **(n=2187)** | **Liver disease cohort**  **(n=758)** |
| --- | --- | --- | --- |
| Initial total variants count | 215,147 | 57,724 | 19,916 |
| Variants: QC filter | 186,818 | 50,443 | 17,884 |
| Unique variants | 31,669 | 14, 319 | 7,202 |
| Pathogenic  HGMD or ClinVar | 1873 | 519 | 208 |
| New Protein Truncating  Variants | 145 | 38 | 16 |
| Individuals carry candidate pathogenic variants | 1577 (20.1%) | 416 (19.0%) | 159(21.0%) |

Supplementary Table 5: Effects of minor allele frequency filtering on the burden of candidate pathogenic variants in genes associated with chronic liver diseases

| MAF* | Candidate  Pathogenic  Variants(n) | Variants Previously Reported as Pathogenic | | | | Protein-Truncating Variants Absent in ClinVar or HMGD at the Time of Analysis | | | |
| --- | --- | --- | --- | --- | --- | --- | --- | --- | --- |
|  |  | Persons, n | Unique SNVs, n | Unique Indels, n | Unique Genes, n | Persons, n | Unique SNVs, n | Unique Indels, n | Unique Genes, n |
| Dominant disorders | | | | | | | | | |
| Total | 322 | 1760 | 248 | 13 | 42 | 138 | 24 | 37 | 28 |
| >1% | 15 | 882 | 14 | 0 | 8 | 50 | 0 | 1 | 1 |
| <1% | 307 | 951 | 234 | 13 | 42 | 88 | 24 | 36 | 28 |
| <0.1% | 245 | 399 | 174 | 13 | 41 | 81 | 24 | 34 | 28 |
| <0.01% | 160 | 124 | 95 | 11 | 37 | 62 | 21 | 33 | 27 |
| Recessive disorders | | | | | | | | | |
| Total | 233 | 343 | 170 | 16 | 54 | 31 | 7 | 40 | 23 |
| Homozygous | 24 | 28 | 19 | 4 | 21 | 1 | 0 | 1 | 1 |
| Compound Heterozygous | | | | | | | | | |
| >1% | 32 | 194 | 30 | 1 | 20 | 1 | 0 | 1 | 1 |
| <1% | 201 | 204 | 140 | 15 | 51 | 30 | 7 | 39 | 22 |
| <0.1% | 132 | 94 | 73 | 13 | 36 | 30 | 7 | 39 | 22 |
